# Retraction: COVID-19 mRNA Vaccines: Lessons Learned from the Registrational Trials and Global Vaccination Campaign

**DOI:** 10.7759/cureus.r137

**Published:** 2024-02-26

**Authors:** M. Nathaniel Mead, Stephanie Seneff, Russ Wolfinger, Jessica Rose, Kris Denhaerynck, Steve Kirsch, Peter A McCullough

**Affiliations:** 1 Biology and Nutritional Epidemiology, Independent Research, Copper Hill, USA; 2 Computer Science and Artificial Intelligence Laboratory, Massachusetts Institute of Technology, Cambridge, USA; 3 Biostatistics and Epidemiology, Independent Research, Research Triangle Park, USA; 4 Immunology and Public Health Research, Independent Research, Ottawa, CAN; 5 Epidemiology and Biostatistics, Independent Research, Basel, CHE; 6 Data Science, Independent Research, Los Angeles, USA; 7 Cardiology, Epidemiology, and Public Health, McCullough Foundation, Dallas, USA; 8 Cardiology, Epidemiology, and Public Health, Truth for Health Foundation, Tucson, USA

The Editors-in-Chief have retracted this article. Following publication, concerns were raised regarding a number of claims made in this article. Upon further review, the Editors-in-Chief found that the conclusions of this narrative review are considered to be unreliable due to the concerns with the validity of some of the cited references that support the conclusions and a misrepresentation of the cited references and available data. The authors disagree with this retraction.

